# Supplementary material for: Experiences of Black and Latinx health care workers in support roles during the COVID-19 pandemic: A qualitative study
Source: PLoS One. 2022 Jan 18;17(1):e0262606. doi: 10.1371/journal.pone.0262606 (PMC8765643; doi:10.1371/journal.pone.0262606)
Supplement: S1 Data — (DOCX) [file pone.0262606.s001.docx]

**Profound impact of the pandemic in job responsibilities, work settings, and connections**

“Well, the impact has been tremendous for me…. I lost my husband during COVID, and I think that - in May, that was kind of the height of the COVID, so I couldn’t do a lot of things. And I still had to work and so forth and take care of the family and try to make them safe.” [Group 1, community health worker in home setting]

“By the middle of April, I’d lost four friends to it… we were altogether Friday, Saturday, Sunday, and they all caught it, and I was spared. I mean, I was spared” (Participant 8, outpatient care staff, hospital setting)

“[O]ur department was actually closed March 20th, and we were put into what they call… a labor pool. So our jobs ranged from either mask distribution, cleaning COVID vents, temperature taking… So a variety of different roles that we played outside of what our normal job duties would be.” [Participant 9, therapy department staff, hospital setting]

“As far as work goes, it’s been implementing new policies every day to the workflow being changed drastically”. [Participant 4, administrative support staff, hospital setting]

“I have a friend of mine that work at the hospital. She works 16 hours. Sixteen hours. She said [name of participant], I barely have no time to eat because she’s standing on her feet to try to help the best way she can. Sometimes they have no equipment in there. They have no gloves, no masks. She said [name of participant], I have to look all over the place to find equipment so I could help taking care of these patient.” [Group 1, certified nurse assistant]

“I’ve also seen that many people have felt like a little bit lost because with all of this involving technology, trying to talk via the internet has been something difficult for the elderly, and communicating with doctors.” [Group 2, community health worker]

“In this city, for example, lead in the paint. We would … go in physically, into the house and we’d see if the paint might have lead…” [Participant 5, community health worker, home care setting]

“And then some residents, they want to get close to you, hold your hand. Their family is not around. They want to talk to you. … You can’t do it no more.” [Participant 7, clinic coordinator, nursing home]

“Human beings cannot only be limited to visual or audio contact… Do you understand me? Physical contact of, hello, a hug, a handshake. I think those things will never be the same after COVID.” [Participant 5, community health worker, home setting]

**Fears and uncertainty caused by the pandemic among HCWs**

“It strikes me how sometimes I get up in the morning. I say I don’t know what I’m going to face now… I have my children. We live together as a family. Sometimes when I come back from work I tell them, you know what? Don’t come close to me. Let me get undressed, take my shower. Then when I’m ready, I’ll come out and then we can say hi to each other. ” [Participant 7, clinic coordinator, nursing home]

“You take care of this today. The next day, by the time you get to work, they told you they have the COVID. They have to move them. Then you say oh, my God. …You don’t protect yourself, but I took care of this person yesterday and then it’s like we are too close to each other.” [Participant 7, clinic coordinator, nursing home]

“There was the fear of saying, yes, I’m positive, because obviously, they would immediately get sent home from work. …And obviously, the falling behind in bills or the fact that you did not work one day, gets you behind with all your bills. For example, rent payment, the electric bill.” [Participant 5, community health worker, home care setting]

“…they were exposed to someone – not at work, but on the ride to get to work. So, the managers tell them, okay, you were exposed outside of work to someone who was positive, so you have to stay home and we’re not going to pay you for those two weeks… they said, no, well, next time, I’m not going to notify them. I’m going to work because if they continue leaving me at home, in quarantine, with no pay, what am I going to do?” (Group 1, community health worker)

“It’s a huge stigma; not from everyone, but some people will make you feel very dirty, very uncomfortable, because someone has tested positive.” [Participant 6, administrative assistant in a hospital setting]

“Right now, two people just came back - three - to our area that were tested positive and were out for a while, and it really gives me the creeps. I don’t know. I’m thinking once it’s in you, it’s in you, and it’s going to get me. So I try to stay as far as I can from them.” [Participant 10, dietary aid, nursing home setting]

**Shifts in testing and vaccines attitudes as the pandemic evolved**

“So it’s basically your choice [testing]. It’s not mandatory. It’s not required.” [Participant 4, administrative support staff, hospital setting]

“…I’d also do it every two weeks on my own behalf because if something happens to me, I didn’t want to affect [my children].” [Group 1, community health worker]

“[S]ometimes I do find it difficult trying to figure out, am I eligible for the free testing that they have, and because I’m not a true employee, should I go somewhere else.” [Participant 6, administrative assistant, hospital setting]

“Ours [test] is twice weekly for the lab, and then they had this thing where we were getting rapid tests three times per week, but that has stopped. …My main concern (…) I don’t know if anybody else see it the way I do, but when it comes to rapid testing, that person is just tested and let go back into their work area until 15 minutes later. I don’t think that is safe because more than once they find people that are positive, and they’ve already gone back to the people that it’s like - it’s - that’s why I’m so scared of it. [Participant 10, dietary aid, nursing home setting]

“I have to keep my parents safe… I was going to say one way of doing that is with testing. The hospital does not test us for COVID. Initially they did. They tested us for COVID. Then they tested us for the antibodies. We had nasal swab, then saliva, and then blood was drawn. And that was done all at once.” [Participant 9, physical therapy department staff, hospital setting]

“Well, I just wanted to see the type of side effects, if there were any, other than just the mild temperature.” [Participant 8, outpatient care staff, hospital setting]

“Initially, it was a hard no… In the very, very beginning, I decided to let my coworkers go first and see what happened with them, and then I would do it. Wednesday evening, I finally logged on to make an appointment…. [Participant 8, outpatient care staff, hospital setting]

“I had questions. And so, part of my questions had to do with testing, believe it or not… Well, I’m not afraid to get it. It’s not that. I have questions and I think it’s fair that…I want my questions answered. So I actually couldn’t get answers from anyone else, so that’s when I wrote the NIH … I felt amazed that they answered me, and so quickly, and very specifically. And then I felt more comfortable about getting the vaccine. [Participant 9, physical therapy department staff, hospital setting]

“Why are they offering it to Newark first? Is it because of the minorities? So they want to experiment on us, right? But then the intelligent part of me says that we should be blessed, and at the same time, if this is a chance to make it go away, then I have to do what I have to do, not for myself, but for my family.” [Participant 4, receptionist, hospital setting]

“Either you are doing it or you are not getting your job. So I don’t think they should - they kind of put us in a place whereby you have to choose between your job and the vaccine.” [Group 2, certified nurse assistant]
