# Supplementary material for: Experiences of Black and Latinx health care workers in support roles during the COVID-19 pandemic: A qualitative study
Source: PLoS One. 2022 Jan 18;17(1):e0262606. doi: 10.1371/journal.pone.0262606 (PMC8765643; doi:10.1371/journal.pone.0262606)
Supplement: Attachment — Submitted filename: Rivera-Nunez_Response_to_Reviewers.docx [file pone.0262606.s002.docx]

**Response to Reviewers**

Reviewer #1

I found the study to be a well-written and much needed investigation to provide specific insight into the perspectives and experiences of this unique population who are both members of a high-risk essential workforce and are also members of marginalized communities that have been most heavily impacted by COVID-19. I found no major issues with this manuscript. Additionally, it is important to note the context/timing of when this study was conducted is significant (Nov 2020-Feb 2021.) It was conducted during a pivotal moment in the pandemic, which coincided with the first rollout of COVID-19 vaccines. The study’s findings successfully captured temporal changes in HCW experiences at the workplace, including testing and evolving perspectives regarding vaccine hesitation. The participants’ responses provided unique insight into many of the barriers that Black and Latinx HCWs, patients and community members alike, encounter with regard to: essential worker workplace challenges, the availability and accessibility of COVID-19 testing and vaccines. The authors’ address various limitations of the study, specifically the limitations of the sample population in that they “purposively sampled only Black and Latinx HCWs…” from largely urbanized counties in one state. Another minor, yet potential limitation is the relatively small (n=17) and homogeneous sample population. However, as stated, this was done intentionally by the authors’ and does indeed capture highly specific data on a very distinct population of “dual perspective” all-female Black and Latinx HCW participants.

1. Another minor issue is in Lines 139-140, referencing Table 1. Line 140 states that 91% percent of the sample population was female. However, Table 1 reflects that of n=17, 100% is female.

**Thank you for identifying this inconsistency in our manuscript. We appreciate the opportunity of correcting it. We have corrected the % and updated the manuscript as follow: Table 1 summarizes demographics and job descriptions of our sample. The median age was 48 years (range 25-58); 47% were Black, 53% Latinx, and 100% female. Lines 140-141**

2. Line 140 also states that 82% of HCWs were Black and 18% were Latinx. Yet, Table 1 reflects that 47.1% were Black and 52.9% were Latinx. Again, these are very minor issues and can be easily revised by the authors. I very much enjoyed reading this manuscript and wish to congratulate the authors on this well-written and thought provoking work.

**Thank you again for identifying this inconsistency in our manuscript. We have corrected the % and updated the manuscript as follow: Table 1 summarizes demographics and job descriptions of our sample. The median age was 48 years (range 25-58); 47% were Black, 53% Latinx, and 100% female. Lines 140-141.**
